# A UAV-based semi-supervised segmentation framework for pine wilt disease via vegetation Index–RGB synergy

**DOI:** 10.1016/j.plaphe.2026.100255

**Published:** 2026-06-27

**Authors:** Tao Liu, Wentao Peng, Huaiqing Zhang, Hui Lin, Langwen Tang, Jingdong Li, Xinyu Zhang, Chaoying He, Zilin Ye, Yingfang Zhu, Guoxiong Zhou, Shichao Jin

**Affiliations:** a Central South University of Forestry and Technology, Changsha, Hunan, 410004, China; b Research Institute of Forest Resource Information, Chinese Academy of Forestry, Beijing, 100091, China; c Henan University of Technology, Zhengzhou, Henan, 450001, China; d Nanjing Agricultural University, Nanjing, Jiangsu, 210014, China

**Keywords:** Pine wilt disease, Canopy phenotyping, Vegetation indices, UAV remote sensing, Semi-supervised semantic segmentation

## Abstract

Pine wilt disease (PWD), caused by the pine wood nematode (Bursaphelenchus xylophilus), continues to threaten forest ecological security. Unmanned aerial vehicle (UAV) remote sensing makes large-scale screening feasible, but accurate canopy segmentation remains difficult in practice. The main obstacles are threefold: (i) canopy appearance changes noticeably from early to late infection stages, which can cause model representations to drift toward later-stage symptoms and weaken subtle early-stage cues; (ii) dense pixel-wise annotation is costly, making semi-supervised learning dependent on imperfect pseudo-labels; and (iii) forest backgrounds are cluttered and often visually similar to diseased regions, limiting the discriminative ability of RGB appearance alone. To address these three practical difficulties, we build a standardized UAV canopy dataset for PWD and develop a lightweight multimodal segmentation framework. The method combines three components. First, Nested-Tempo Memory Consolidation (NTMC) is designed as a stage-aware extension of EMA-based teacher–student learning. It maintains nested fast/medium/slow temporal trajectories to retain stage-specific knowledge while improving cross-stage stability during Early–Middle–Late sequential training. Second, Drift-Compensated Consistency Regularization (DCCR) integrates reliability calibration into semi-supervised consistency learning, so that unlabeled samples contribute training signals mainly when they are sufficiently reliable, reducing error accumulation from noisy pseudo-supervision. Third, Vegetation-index-conditioned Cross-Modal Attention (VCCA) uses vegetation indices—Normalized Difference Vegetation Index (NDVI) and Enhanced Vegetation Index (EVI)—as physiological cues to modulate visual features, thereby reducing the dependence on RGB appearance and improving feature discrimination under texture-similar forest backgrounds. Experiments on one in-house dataset and three external datasets show consistent improvements in mean intersection over union (mIoU), F1-score, and Matthews correlation coefficient (MCC). With all components enabled, the framework improves mIoU from 0.6015 to 0.6829 over the baseline and produces cleaner disease boundaries with fewer background false alarms, demonstrating its potential for practical UAV-based forest disease monitoring.

## 1 Introduction

Pine wilt disease progresses rapidly and can trigger widespread pine dieback, reshaping stand structure and weakening forest functions within a single season. From an ecological perspective, the concern is not only which trees are symptomatic, but also how far the infection has advanced within and across crowns, because canopy damage directly affects photosynthetic capacity, transpiration, habitat quality, and ultimately carbon and water regulation at the stand level [[1], [2], [3]].

Fine-grained crown segmentation therefore serves as a phenotyping tool: it delineates diseased tissue and the gradual transition from healthy to stressed canopy, allowing managers to quantify affected crown area more objectively, track disease expansion in space, and prioritize sanitation felling or quarantine in a way that minimizes collateral disturbance while containing spread [4,5]. In conventional field practice, the most common approaches rely on manual inspection and plot-based surveys. Although these methods are intuitive, they are labor-intensive, offer limited spatial coverage, and depend heavily on the experience of the surveyors [6].

Traditional image-processing techniques—such as thresholding, edge detection, and morphological operations—have also been used to extract canopy regions or lesion boundaries. However, under shadows, understory vegetation, or soil textures that resemble diseased areas, these methods often misidentify boundaries and produce unreliable delineations [7]. As a result, these methods tend to become unstable once the scene changes—e.g., variations in illumination, background conditions, or canopy overlap—making them difficult to scale to large-area and continuous monitoring [8].

To ease the burden of manual interpretation, early work often relied on conventional automated workflows that couple handcrafted cues—e.g., color, texture, and simple shape descriptors—with classical classifiers such as SVMs or random forests to separate diseased from healthy crowns or to obtain coarse lesion maps [9,10]. In parallel, some studies adopted unsupervised or weakly supervised segmentation routines, including clustering, region growing, watershed transforms, and graph-cut formulations, to replace manual delineation. In practice, these methods are easily disturbed by illumination changes, background clutter, and within-crown heterogeneity, and their outcomes depend strongly on parameter choices and initialization. As a result, the generated masks often break into disconnected fragments, and boundary placement can vary noticeably from one image to another [[11], [12], [13]]. Overall, this line of work can improve efficiency, but achieving stable and fine boundary delineation in complex forest environments remains challenging. Consequently, end-to-end learning-based methods have become increasingly popular [14].

**Fig. 1:**
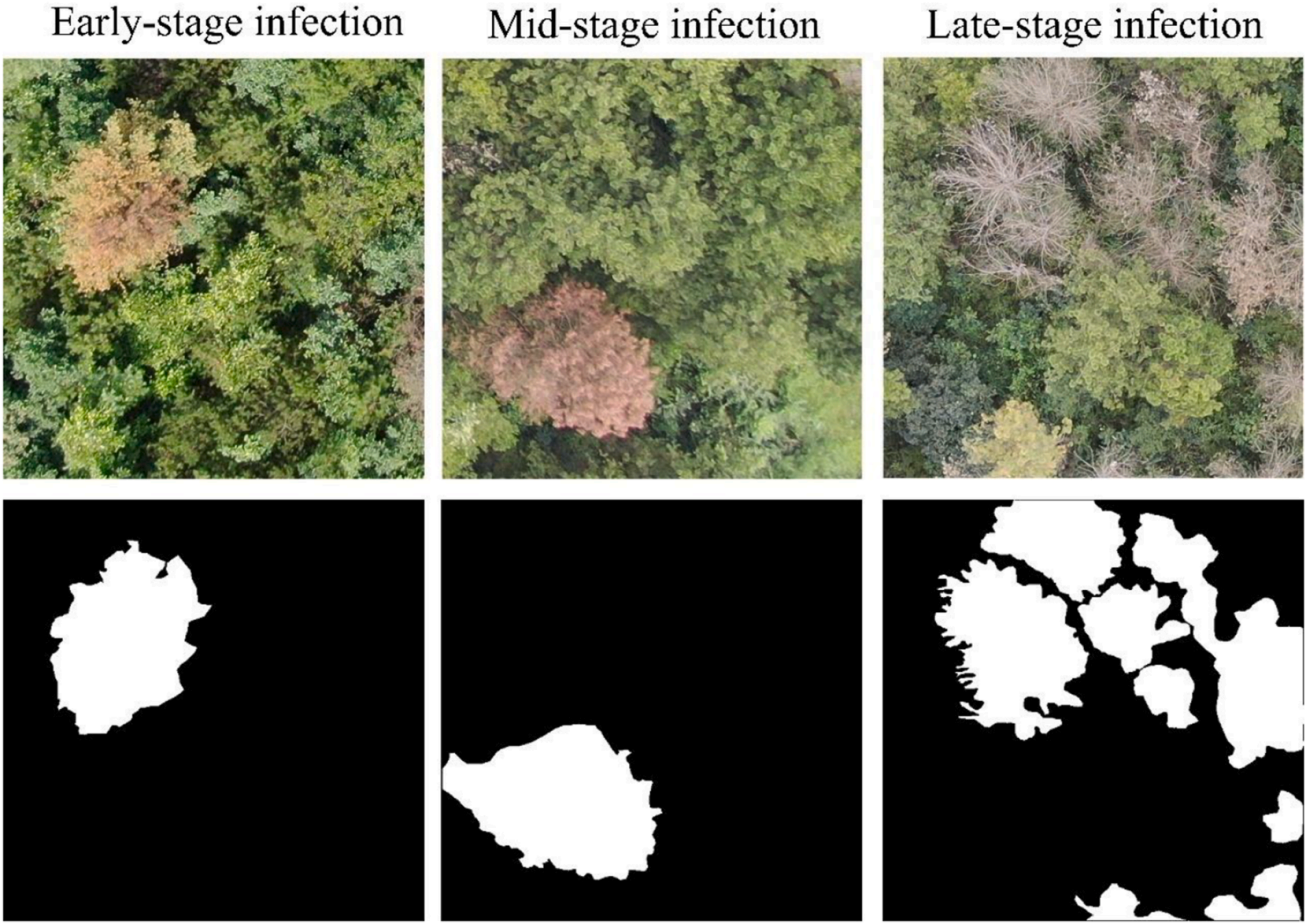
Pine infection at different stages. The top row shows the original images, and the bottom row shows the corresponding labels.
2) Costly and variable-quality canopy labeling. Fine-grained disease segmentation depends on dense pixel-level annotations, but delineating diseased crowns and transition zones is labor-intensive and can vary across annotators, especially under blurred boundaries and background interference. With limited high-quality labels, supervised models are more likely to fit the training set too tightly and lose robustness when conditions change [17].
3) Relying on RGB imagery alone provides only a partial view of canopy phenotypes in pine wilt disease. Visible-color appearance is easily confounded by illumination geometry, shadows, understory/background variation, and sensor conditions, while key physiological changes (e.g., water status, pigment dynamics, and stress-related reflectance shifts) are not consistently expressed in RGB space. As a result, RGB-only recognition can become unreliable in texture-similar forest backgrounds. Integrating plant-physiology–related modalities, such as vegetation indices derived from multispectral data, including NDVI and EVI, can provide complementary cues for more discriminative canopy segmentation [4].

With the rapid development of artificial intelligence, deep learning has been increasingly adopted for pine wilt disease segmentation in recent years. A major reason is that it can learn discriminative representations directly from data, reducing the reliance on handcrafted rules. For example, U-Net–style semantic segmentation networks have been applied to UAV imagery to perform pixel-wise delineation of diseased canopies, enabling automatic separation of infected and healthy regions [4]. In addition, instance segmentation frameworks such as Mask R-CNN have been introduced to UAV RGB imagery to jointly localize infected trees and extract diseased canopy regions, supporting fine-grained monitoring of pine wilt disease [15]. However, mainstream deep-learning approaches in this area still face several prominent challenges:

1) As shown in Fig. 1, across infection stages, pine wilt disease expresses distinct canopy phenotypes. In practice, models are often drawn to the most obvious late-stage discoloration and dieback, while the subtler early and transitional phenotypes are learned less reliably, which can result in stage-unstable recognition [16].

The symptoms of pine wilt disease vary substantially from the early to middle and late stages, making sequential training particularly prone to multi-stage feature forgetting. To mitigate this issue, Gao and Liu improved knowledge retention by estimating which parameters are more critical to previously learned information [18]; however, such approaches typically require additional importance estimation or approximate computations. Wen et al. alleviated forgetting caused by conflicts between new and old knowledge by distilling diverse information from multiple complementary teachers into a student model [19]; however, when samples from earlier stages are scarce or early symptoms are extremely subtle, performance can still degrade. Behrouz et al. further characterized the learning process as a set of nested, multi-level optimization mechanisms [20]; yet, when extended to dense prediction tasks such as pixel-wise segmentation, this paradigm often needs to be tightly integrated with the specific network architecture and training pipeline, which increases implementation complexity.

To address the scarcity and high cost of high-quality annotations, a straightforward solution is to adopt fully supervised semantic segmentation frameworks, such as U-Net, DeepLab and their variants, and train models with sufficiently annotated data. These methods can achieve high accuracy when annotations are abundant and the data distribution is relatively consistent; however, their performance strongly depends on large-scale, fine-grained, and consistently labeled masks. As data collection expands to more diverse real-world settings, small-sample annotation has emerged as an active direction for reducing labeling cost. In contrast, semi-supervised segmentation leverages a small set of high-quality labeled samples together with a large amount of unlabeled data to effectively enlarge the training set without substantially increasing annotation effort. This paradigm has therefore become increasingly important for pine wilt disease segmentation [[21], [22], [23]].

Semi-supervised segmentation compensates for limited supervision by exploiting abundant unlabeled data, allowing models to learn more stable discriminative features under scarce annotations. Yang et al. systematically strengthened the weak-to-strong consistency paradigm [24]; however, this line of work remains sensitive to the choice of augmentation strategies and confidence-based pseudo-label filtering. Fang et al. improved the usability of unlabeled supervision by identifying unreliable regions with an uncertainty map and applying targeted denoising [25]; however, uncertainty estimation and noise localization themselves introduce additional complexity. Liu et al. provided a more rigorous analysis of pseudo-label selection and proposed improved strategies that explicitly increase the separability between reliable and unreliable predictions, thereby enhancing the stability of semi-supervised segmentation [26]; nevertheless, in forestry imagery where cross-scene distribution shifts are pronounced, reliability assessment can still fluctuate under domain drift. As a result, the training schedule and thresholding/separation strategies often require careful tuning to remain effective across domains.

To reduce reliance on RGB appearance and strengthen physiological cues, Yu et al. leveraged UAV multispectral imagery and stratified pine wilt disease by infection stages, showing that multispectral cues can be particularly beneficial for detecting subtle early-stage symptoms [27]. However, multispectral acquisition and radiometric calibration impose stricter requirements. Xu et al. further developed a UAV multispectral detection framework built upon an improved YOLOv8 architecture [12,13]. Nevertheless, these studies are mostly detection-oriented and do not directly address pixel-level delineation, which typically demands stronger fusion and decoding designs. At the regional scale, Long et al. exploited long-term multi-source remote-sensing time series and incorporated vegetation indices such as NDVI to support PWD monitoring and forecasting [28]; however, region-scale approaches typically cannot directly produce fine-grained, tree-level or canopy-level segmentation results.

Overall, introducing vegetation indices makes physiological differences—previously implicit in multispectral bands—more explicit. In particular, PWD-induced changes such as chlorophyll decline and structural degradation can be transformed into features that are more stable and separable, which helps reduce RGB-based ambiguity caused by texture-similar forest backgrounds and unstable visual appearance.

Existing studies have provided useful directions from three perspectives: anti-forgetting learning, semi-supervised training, and multimodal information integration. However, most methods advance along a single axis and therefore resolve only part of the problem. Knowledge-retention–oriented approaches may struggle to maintain stage adaptivity; semi-supervised strategies can be undermined by the accumulation of noisy pseudo-labels; and index-assisted cues, if not tightly coupled with a lightweight segmentation architecture, are often underutilized. For stage-aware segmentation of pine wilt disease, a practical framework is needed to jointly consider stage-wise drift, unreliable pseudo-label supervision, and RGB-based background confusion in real, complex forest environments. To address this need, this study presents the first UAV-based semi-supervised semantic segmentation framework for pine wilt disease canopy phenotyping.

Inspired by the above observations, this paper presents NDVNet, a task-driven collaborative segmentation framework for UAV-based pine wilt disease canopy segmentation. Rather than positioning NDVNet as a universal visual learning paradigm, we define it as a lightweight collaborative framework for canopy-level phenotypic segmentation of PWD in UAV remote-sensing imagery. The method builds upon a lightweight segmentation backbone and incorporates multimodal inputs to support robust segmentation under stage-wise phenotypic shifts and limited pixel-level annotations. The main contributions are summarized as follows:

(1) PWD canopy phenotyping dataset. We construct a standardized UAV-based canopy segmentation dataset for pine wilt disease, with support from the Hunan Academy of Forestry. The dataset contains pixel-level annotations and unlabeled samples across early, middle, and late infection stages, providing a practical basis for stage-aware and semi-supervised PWD canopy segmentation.
(2) Stage-aware and annotation-efficient learning. Stage-wise phenotypic changes can cause the model representation to drift toward later-stage symptoms, which may further lead to the forgetting of subtle early-stage cues. To alleviate this issue, we introduce NTMC as a task-driven extension of EMA-based teacher–student learning. NTMC maintains nested fast/medium/slow temporal trajectories during early–middle–late sequential learning, aiming to balance current-stage adaptation with previous-stage retention. To reduce the influence of unreliable pseudo-labels, we further introduce DCCR, which uses reliability-weighted consistency learning to exploit unlabeled images more reliably.
(3) Vegetation-index-guided cross-modal calibration. To reduce background confusion caused by the limited discriminative ability of RGB appearance, we incorporate NDVI and EVI through VCCA. The vegetation-index cues provide complementary physiological information for RGB features, helping the model distinguish diseased canopy regions from texture-similar forest backgrounds.

## 2 Study area and data collection

The UAV-based pine wilt disease (PWD) canopy remote-sensing imagery used in this study was provided by the Hunan Academy of Forestry, China. The data were collected in 2024 using a Zhonghang CW-15 UAV flying at an altitude above 500 m, resulting in a ground sampling distance (GSD) of approximately 0.10 m. RGB imagery was acquired using a 61-megapixel full-frame camera equipped with a 35 mm focal-length lens. Meanwhile, to support vegetation index construction, multispectral imagery was synchronously collected using a MicaSense RedEdge-P multispectral sensor, with bands including Blue (475 ± 32 nm), Green (560 ± 27 nm), Red (668 ± 16 nm), Red-edge (717 ± 12 nm), and Near-infrared (842 ± 57 nm), and was further processed into georeferenced orthomosaics. Radiometric calibration was performed using a downwelling light sensor (DLS 2) together with a calibrated reflectance panel to obtain reflectance products. After orthorectification and radiometric preprocessing, NDVI, EVI, and other vegetation index (VI) values were calculated from the multispectral reflectance bands. These vegetation-index features were then mapped to the corresponding sample regions and combined with RGB imagery to construct multimodal inputs for subsequent model fusion learning. To ensure spatial consistency among different modalities, the relevant vegetation-index results were resampled under a unified spatial reference using bilinear interpolation. The surveyed sites are located in several representative forest regions of Hunan Province, including Anren, Xiangjiang New Area, Jiuyi Mountain, and Tianhua Mountain, with an overall geographic extent of approximately 25.26°N–28.50°N and 111.96°E−113.28°E. The flight campaigns were conducted from May to June 2024 (late spring to early summer) to capture representative canopy phenotypic variations of PWD during this period.

The surveyed forest area covers approximately 1174.5 km^2^. Given that high-altitude imagery typically has a large scene footprint and exhibits non-uniform target density, we cropped and screened the raw orthomosaic imagery to construct the dataset. In total, 848 RGB image tiles containing multiple diseased pine canopies were retained, including 431 labeled tiles with pixel-wise annotations and 417 unlabeled tiles for semi-supervised learning. All tiles (and their aligned VI maps) were uniformly resized to 640×640 before being fed into the network. Furthermore, to support continual learning, all tiles were categorized into three disease-progression stages (Early/Middle/Late), resulting in 160/340/348 tiles, respectively. The labeled tiles were distributed across stages proportionally (81/173/177 for Early/Middle/Late), while the remaining tiles in each stage were treated as unlabeled (79/167/171 for Early/Middle/Late). For each stage, validation (10%) and test (15%) sets were sampled exclusively from labeled tiles, and the remaining tiles were used for training (≈75%); unlabeled tiles were used only in the training stage for the unsupervised branch. Concretely, the Early stage includes 24 test tiles and 16 validation tiles, leaving 120 training tiles (41 labeled + 79 unlabeled); the Middle stage includes 51 test tiles and 34 validation tiles, leaving 255 training tiles (88 labeled + 167 unlabeled); and the Late stage includes 52 test tiles and 35 validation tiles, leaving 261 training tiles (90 labeled + 171 unlabeled). Continual training was performed sequentially in the biological order (Early→Middle→Late), and all stage-specific test sets were strictly held out from training and model selection to simulate real-world disease monitoring and evaluate continual learning capability.

## 3 Methods

This work presents a lightweight multimodal network for canopy-level phenotyping and segmentation of pine wilt disease (PWD) from UAV imagery. RGB images are used as the main source of spatial detail, while vegetation indices (e.g., NDVI and EVI) are incorporated as complementary cues that better reflect canopy physiological status. Within an encoder–decoder framework, the index signals are introduced through a cross-modal conditional calibration step, so that visual features are adjusted according to vegetation-index responses. This design is intended to reduce the dependence on RGB appearance and improve feature discrimination under complex forest backgrounds.

**Fig. 2:**
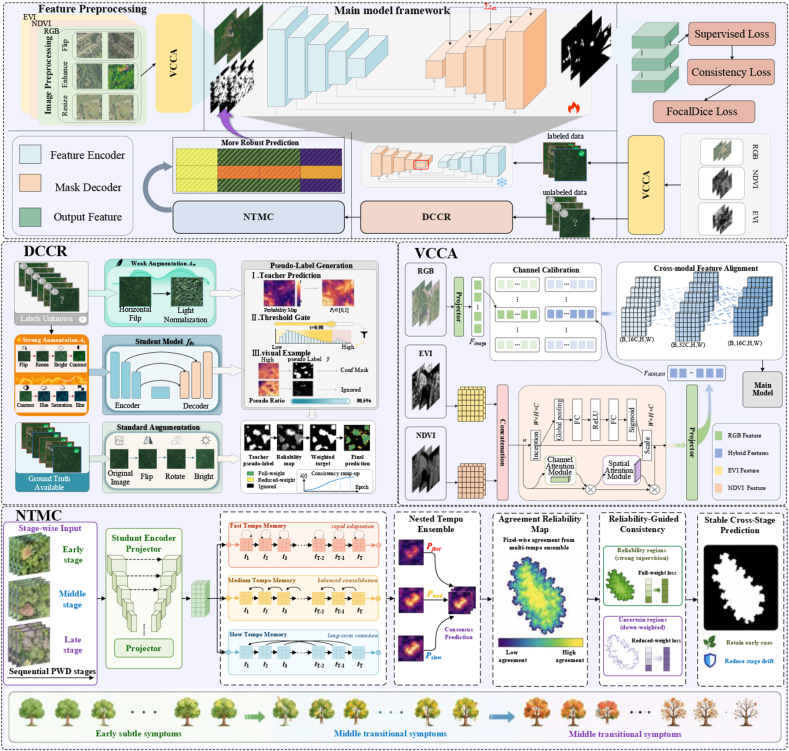
Overall framework and workflow of NDVNet.

Model training is organized to match two practical constraints in PWD monitoring: stage-dependent phenotypes and limited high-quality labels. For sequential early/middle/late stages, we adopt nested fast/middle/slow tempo updates to retain information from subtle and transitional phenotypes while reducing the tendency of the model to drift toward only the most obvious late-stage symptoms. Under scarce annotations, we employ consistency learning so that unlabeled images can contribute useful supervision, and we couple it with reliability calibration to reduce the influence of noisy pseudo-labels. The overall training and optimization procedure is summarized in Algorithm 1, and the corresponding schematic is shown in Fig. 2.

### 3.1 NTMC

NTMC is not intended to replace the conventional EMA-based teacher–student paradigm. Instead, it is designed as a stage-aware structural extension of this paradigm for sequential PWD canopy segmentation. Different from a single EMA teacher that maintains only one smoothed trajectory, NTMC organizes fast, medium, and slow EMA trajectories into a nested temporal hierarchy. The fast trajectory responds more rapidly to current-stage phenotypic changes, the medium trajectory balances adaptation and retention, and the slow trajectory provides a more stable cross-stage reference. Therefore, the methodological contribution of NTMC lies not in the isolated use of EMA itself, but in the drift-aware nested organization of multiple temporal trajectories for stage-wise consolidation.

To make the update process explicit, all three tempo branches are initialized from the same student model before sequential training starts, with $\phi_{0}^{\left(f\right)}=\phi_{0}^{\left(m\right)}=\phi_{0}^{\left(s\right)}=\theta_{0}$. During training, only the student network is updated by back-propagation on the current mini-batch, while the fast-, medium-, and slow-tempo branches are updated by exponential moving average after each iteration. The update rule is defined as:

(1) $$\begin{array}{c} \phi_{t}^{\left(k\right)}=\alpha_{t}^{\left(k\right)}\phi_{t-1}^{\left(k\right)}+\left(1-\alpha_{t}^{\left(k\right)}\right)\theta_{t},k\in \left\{f,m,s\right\} \end{array}$$

Here, $\theta_{t}$ denotes the student parameters after the current iteration, $\phi_{t}^{\left(k\right)}$ denotes the parameters of the k-th tempo branch, and $\alpha_{t}^{\left(k\right)}$ is the corresponding temporal retention coefficient. In the fixed-tempo setting, the fast, medium, and slow branches use EMA decay factors of 0.90, 0.99, and 0.999, respectively. Thus, the fast branch follows the current student more closely, the medium branch provides a balanced temporal reference, and the slow branch preserves longer-term cross-stage information.

In full NTMC, the fast, medium, and slow branches are further treated as a nested drift-aware hierarchy rather than only fixed labels. The drift indicator is defined as $\delta_{t}={\left\|\theta_{t}-\theta_{t-1}\right\|}_{2}$, and the gradient norm $\left\|g_{t}\right\|$ can also be used as an alternative drift measure. The instantaneous retention coefficient is defined as:

(2) $$\begin{array}{c} \alpha_{t}^{\left(k\right)}=\sigma \left(\eta_{k}\left(\mu_{k}-\delta_{t}\right)\right),k\in \left\{f,m,s\right\} \end{array}$$

where σ(·) is the Sigmoid function, $\mu_{k}$ controls the drift-response threshold, and $\eta_{k}$ controls the response slope. To enforce a strict nested ordering from fast to medium to slow, we parameterize the thresholds as:

(3) $$\begin{array}{c} \mu_{f}=\mu_{0},\mu_{m}=\mu_{0}+\exp\left(\Delta_{1}\right),\mu_{s}=\mu_{0}+\exp\left(\Delta_{1}\right)+\exp\left(\Delta_{2}\right) \end{array}$$

This parameterization guarantees $\mu_{f}<\mu_{m}<\mu_{s}$ and maintains $\alpha_{t}^{\left(f\right)}<\alpha_{t}^{\left(m\right)}<\alpha_{t}^{\left(s\right)}$, yielding a nested short-/mid-/long-term consolidation hierarchy. Therefore, the fast branch has a shorter effective temporal scale and adapts more rapidly to current-stage changes, whereas the slow branch has a longer effective temporal scale and contributes more to cross-stage memory retention.

For an unlabeled sample $x_{u}$, the three tempo branches generate prediction maps from the weakly augmented view:

(4) $$\begin{array}{c} p_{k}\left(x_{u}\right)=\sigma \left(g_{\phi^{\left(k\right)}}\left(A_{w}\left(x_{u}\right)\right)\right),k\in \left\{f,m,s\right\} \end{array}$$

To obtain a stable cross-stage target, we form a weighted ensemble of the multi-tempo outputs:

(5) $$\begin{array}{c} p_{\text{ens}}\left(x_{u}\right)=\sum_{k\in \left\{f,m,s\right\}}\omega_{k}p_{k}\left(x_{u}\right),\sum_{k\in \left\{f,m,s\right\}}\omega_{k}=1 \end{array}$$

The ensemble target is detached from gradient back-propagation and used as the teacher target for consistency learning. Rather than introducing extra replay data, NTMC further uses the agreement among the three tempo predictions as a pixel-level reliability cue. Pixels where the fast/medium/slow outputs agree are typically those with more stable canopy signals, so a stronger consistency constraint is imposed. In contrast, large disagreement tends to occur around weak or emerging symptoms, mixed canopy conditions, and fuzzy transition zones. Supervision in these regions is automatically down-weighted to avoid locking in unreliable pseudo-labels and carrying them forward across stages.

The nested-tempo consistency loss is defined as:

(6) $$\begin{array}{c} L_{\text{NTMC}}=\frac{\sum_{i\in \Omega }M_{i}r_{i}\;l\left(p_{\theta }{\left(A_{s}\left(x_{u}\right)\right)}_{i},p_{\text{ens}}{\left(x_{u}\right)}_{i}\right)}{\sum_{i\in \Omega }M_{i}+\varepsilon } \end{array}$$

Here, $A_{w}\left(\cdot \right)$ and $A_{s}\left(\cdot \right)$ represent weak and strong augmentations, respectively; $M_{i}$ is the reliability-gating mask at pixel i; $r_{i}$ is the agreement-based reliability weight; l(·) can be instantiated as MSE on logits or KL divergence; Ω denotes the set of pixels; and ε is a small constant to avoid division by zero. Finally, the student parameters are optimized jointly by the supervised segmentation loss and the nested-tempo consistency regularization, where λ(t) is used to schedule the contribution of $L_{\text{NTMC}}$ during training. In this way, the nested tempos provide both a cross-stage reference state and a practical reliability signal, improving robustness to forgetting and drift during continual learning of stage-dependent canopy phenotypes.

### 3.2 DCCR

To reduce the dependence on dense, pixel-level labels—which are costly to obtain for canopy disease mapping—we employ a semi-supervised strategy that combines teacher–student consistency learning with reliability-aware weighting. In this setup, the student is trained to produce stable segmentation maps under strong perturbations, while the teacher provides smoother soft targets from weakly augmented views. Unlabeled images contribute to training by encouraging the student to match the teacher, as summarized in the DCCR block of Fig. 2.

Because PWD crowns often show blurred transition zones and complex background interference, especially at early stages when symptoms are weak, pseudo-labels are not equally trustworthy across the image. We therefore introduce a reliability calibration step to convert teacher confidence and prediction agreement into pixel-wise weights. Highly reliable regions are assigned the full consistency weight, uncertain but still informative regions are assigned a reduced weight, and unreliable regions are ignored in the unlabeled loss. This prevents noisy pseudo-labels, which are often concentrated around ambiguous boundaries or weak-stress areas, from being reinforced and amplified during training.

For an unlabeled sample $x_{u}$, the student prediction under strong augmentation and the teacher target under weak augmentation are defined as:

(7) $$\begin{array}{c} P_{i}=\sigma {\left(g_{\theta_{s}}\left(A_{s}\left(x_{u}\right)\right)\right)}_{i},Q_{i}=\text{stopgrad}\left[\sigma {\left(g_{\theta_{t}}\left(A_{w}\left(x_{u}\right)\right)\right)}_{i}\right] \end{array}$$

Here, $\theta_{s}$ and $\theta_{t}$ denote the parameters of the student and teacher networks, respectively; g(·) outputs the network logits; σ(·) denotes the Sigmoid/Softmax activation; $A_{w}\left(\cdot \right)$ and $A_{s}\left(\cdot \right)$ denote weak and strong augmentations; and i indexes a pixel location. The stop-gradient operation prevents the teacher target from being updated by back-propagation.

More specifically, the confidence score of each pixel is computed from the teacher probability distribution as the maximum class probability. When NTMC is enabled, we further use the agreement among the fast-, medium-, and slow-tempo teacher predictions as an additional reliability cue. The final pixel-level reliability score is defined as:

(8) $$\begin{array}{c} c_{i}=\max_{c}Q_{i,c},\rho_{i}=c_{i}\cdot a_{i} \end{array}$$

Here, $c_{i}$ denotes the teacher confidence at pixel i, and $a_{i}$ denotes the multi-tempo agreement score. If NTMC is not enabled in an isolated DCCR experiment, $a_{i}$ is set to 1, so the reliability score is determined by teacher confidence only. Based on the reliability score $\rho_{i}$, we define the reliability mask and weight as:

(9) $$M_{i}=1\left(\rho_{i}\geq 0.90\right),r_{i}=\left\{ \begin{array}{cc} 1.0, & \rho_{i}\geq 0.98\\ 0.3, & 0.90\leq \rho_{i}<0.98\\ 0, & \rho_{i}<0.90 \end{array}\right.$$

Thus, pixels with $\rho_{i}\geq 0.98$ are regarded as highly reliable regions and receive the full consistency weight. Pixels with $0.90\leq \rho_{i}<0.98$ are treated as uncertain but still informative regions and receive a reduced weight. Pixels with $\rho_{i}<0.90$ are ignored in the unlabeled loss. In this way, DCCR does not directly trust all pseudo-labels, but selectively uses them according to their pixel-wise reliability.

With the reliability weight and gating mask, the unlabeled consistency loss is defined as:

(10) $$\begin{array}{c} L_{u}=\frac{\sum_{i\in \Omega }M_{i}r_{i}\;l\left(P_{i},Q_{i}\right)}{\sum_{i\in \Omega }M_{i}+\varepsilon } \end{array}$$

Here, l(·) can be instantiated as MSE on logits or KL divergence, Ω denotes the set of pixels, and ε is a small constant to avoid division by zero. To reduce the impact of unreliable pseudo-labels in the early training stage, we apply a progressively increasing weight schedule to the unlabeled term. The final training objective of DCCR is written as:

(11) $$\begin{array}{c} L=L_{\sup}+\lambda_{\max}r_{up}\left(t\right)L_{u} \end{array}$$

Here, Lsup denotes the supervised segmentation loss on labeled samples, rup(t) is a ramp-up function that increases monotonically with the epoch or iteration t, and $\lambda_{\max}$ denotes the maximum weight assigned to the unlabeled term. Through this closed-loop design—generating targets from the weak view, learning invariance from the strong view, denoising via reliability calibration, and stabilizing convergence via weight scheduling—the model can exploit unlabeled data more reliably under scarce annotations and consequently improve segmentation performance.

### 3.3 VCCA

To reduce over-dependence on RGB appearance and improve feature discrimination under complex forest backgrounds, we use vegetation indices, including NDVI and EVI, as conditioning cues to guide the recalibration of RGB features. The motivation is practical: RGB imagery carries rich spatial details for canopy edges and fine textures, but it can be disturbed by soil color, shadows, understory vegetation, and acquisition conditions. Vegetation indices, in contrast, more directly reflect changes related to canopy vigor and stress, making them useful for anchoring the interpretation of uncertain visual patterns.

Vegetation-index construction and spatial normalization. Multispectral imagery containing the near-infrared band was acquired synchronously with RGB during the same flight campaign, as described in Section 2. After radiometric calibration and orthorectification, NDVI and EVI were computed from the multispectral reflectance products as:

(12) $$\text{NDVI}=\frac{\rho_{\text{NIR}}-\rho_{R}}{\rho_{\text{NIR}}+\rho_{R}+\epsilon },\text{EVI}=G\cdot \frac{\rho_{\text{NIR}}-\rho_{R}}{\rho_{\text{NIR}}+C_{1}\rho_{R}-C_{2}\rho_{B}+L+\epsilon }\text{.}$$

where $\rho_{B}$, $\rho_{R}$, and $\rho_{NIR}$ denote the radiometrically preprocessed blue, red, and near-infrared reflectance, respectively, and ε is a small constant for numerical stability. We use the standard parameters G=2.5, $C_{1}=6$, $C_{2}=7.5$, and L=1. The NDVI and EVI maps are stacked as a two-channel vegetation-index input, normalized to a consistent range, and resized to match the RGB spatial grid and GSD, so that pixel-wise correspondence is preserved.

In implementation, the aligned NDVI and EVI maps are passed through a lightweight vegetation-index encoder to obtain a compact representation Z. The encoder consists of two consecutive 3×3 convolutional blocks, and each block contains convolution, batch normalization, and ReLU activation. A 1×1 convolution is then used to project the encoded vegetation-index feature to the same channel dimension as the corresponding RGB feature map. In this work, VCCA is inserted at the middle encoder stage, where the feature resolution still preserves useful spatial details while containing stronger semantic information than shallow features.

After feature alignment, Z is used to generate channel-wise and spatial modulation weights. The channel modulation first recalibrates the RGB feature responses according to vegetation-index evidence, and the spatial modulation then emphasizes regions with consistent index-supported disease cues. As a result, regions supported by stable vegetation-index responses are encouraged to activate more strongly, while areas with weak or unstable index responses are suppressed. As shown in the VCCA module of Fig. 2, the calibrated features are finally fused with the original RGB semantic features and forwarded to the decoder, allowing the model to combine index-level physiological cues with image-level structural details. The corresponding formulation is given as follows:

(13) $$\begin{array}{c} F^{c}=a⊙F \end{array}$$

Here, ⊙ denotes element-wise multiplication with channel-wise broadcasting, and $F^{c}$ is the channel-calibrated visual feature.

(14) $$\begin{array}{c} S=Concat\left(AvgPool_{c}\left(F^{c}\right),MaxPool_{c}\left(F^{c}\right),AvgPool_{c}\left(Z\right)\right) \end{array}$$

Here, $AvgPool_{c}$ and $MaxPool_{c}$ denote channel-wise average and max pooling, respectively. *S* aggregates spatial evidence that jointly reflects visual saliency and index-driven trends.

(15) $$\begin{array}{c} b=\sigma \left(Conv_{k\times k}\left(S\right)\right),b\in R^{1\times H\times W} \end{array}$$

(16) $$\begin{array}{c} F^{out}=Fuse\left(F^{cs}\right)=Conv_{1\times 1}\left(Concat\left(F^{\text{cs}},Z\right)\right) \end{array}$$

Here, $F^{out}$ denotes the final multimodally calibrated representation, which is fed into the subsequent decoder to produce the segmentation output.

## 4 Experiment and analysis

### 4.1 Experimental setup and implementation details

All experiments were performed on an Ubuntu 20.04 workstation with two Intel Xeon Platinum 8375 CPUs (2.9 GHz), 72 GB RAM, and an NVIDIA RTX 3090 GPU. The software environment included Python 3.8, PyTorch 2.0.0, and CUDA 11.8. To ensure fair comparisons, all experiments were conducted under the same hardware and software settings.

The input RGB images and the corresponding vegetation-index maps were resized to 640 × 640. All models were trained using the AdamW optimizer with an initial learning rate of 1 × 10^−4^, a batch size of 8, and a weight decay of 1 × 10^−4^. A cosine learning-rate schedule was adopted, and the total number of training epochs was set to 300. The best checkpoint was selected according to the validation-set mIoU and then evaluated on the held-out test set.

For data augmentation, standard augmentation was applied to labeled samples, including random horizontal flipping with a probability of 0.5, random rotation within ±15°, brightness adjustment within 0.8–1.2, and normalization. For unlabeled samples, weak augmentation included random flipping and normalization, while strong augmentation further included random rotation, brightness/contrast perturbation within 0.8–1.2, and Gaussian blur. All geometric transformations were applied consistently to images and masks to maintain pixel-level alignment. The dataset was split in a stage-stratified manner: for each infection stage, 10% of labeled samples were used for validation, 15% for testing, and the remaining samples for training. Unlabeled samples were used only in the semi-supervised training branch and were excluded from validation and testing.

For the loss configuration, the supervised segmentation loss was implemented as FocalDice loss. The consistency loss was implemented as KL divergence between the student prediction and the teacher target. The maximum consistency weight was set to 1.0, and the full and reduced pixel-level consistency weights in DCCR were set to 1.0 and 0.3, respectively. These settings were kept fixed across all experiments unless otherwise specified.

To further ensure the fairness of baseline comparisons, all compared methods were trained and evaluated under a unified experimental protocol. Specifically, the same training, validation, and test splits, input resolution, optimizer, batch size, learning-rate schedule, number of epochs, and evaluation metrics were used for all methods. The input modalities were kept consistent across the compared methods. RGB images were used as the common visual input, while vegetation-index information (NDVI/EVI) was provided only to methods that were explicitly designed to support multimodal fusion. For pretrained weights, all methods followed the initialization strategy reported in their original implementations, and no additional pretrained models or external datasets were introduced for the proposed method. The data augmentation strategy was also kept consistent, with identical labeled-sample augmentations and the same weak/strong augmentation pipeline for semi-supervised training. Furthermore, the labeled/unlabeled split, pseudo-label generation protocol, consistency-loss configuration, and evaluation procedure were kept identical across semi-supervised baselines whenever applicable. Therefore, the reported performance differences mainly reflect the effectiveness of the compared learning strategies rather than inconsistencies in experimental settings.

### 4.2 Evaluation metrics overview

To comprehensively assess segmentation performance, following prior studies in Plant Phenomics and related plant phenotyping/segmentation works, we report five metrics: mIoU, Precision, F1-score, Recall, and MCC [[29], [30], [31]]. Here, TP denotes pixels correctly predicted as foreground (diseased canopy), FP denotes background pixels incorrectly predicted as foreground, FN denotes foreground pixels incorrectly predicted as background, and TN denotes pixels correctly predicted as background.

(1) Mean Intersection over Union (mIoU). Measures the average overlap between the predicted regions and the ground truth, reflecting overall segmentation accuracy.

(17) $$\begin{array}{c} \text{mIoU}=\frac{1}{C}\;\sum_{C=1}^{C}\frac{TP_{c}}{TP_{c}+FP_{c}+FN_{c}} \end{array}$$

(2) Precision. Measures how reliable the predicted foreground pixels are, indicating the degree of false-positive suppression.

(18) $$\begin{array}{c} \text{Precision}=\frac{TP}{TP+FP} \end{array}$$

(3) F1-score. A single summary metric that balances Precision and Recall, suitable when both false positives and false negatives matter.

(19) $$\begin{array}{c} F1-\text{score}=\frac{2\;\text{Precision}\cdot \text{Recall}}{\text{Precision}+\text{Recall}} \end{array}$$

(4) Recall. Measures how completely the ground-truth foreground is retrieved, indicating the degree of false-negative reduction.

(20) $$\begin{array}{c} \text{Recall}=\frac{TP}{TP+FN} \end{array}$$

(5) Matthews Correlation Coefficient (MCC). A correlation-based measure that jointly considers TP/FP/TN/FN and remains informative under class imbalance.

(21) $$\begin{array}{c} \text{MCC}=\frac{TP\cdot TN-FP\cdot FN}{\sqrt{\left(TP+FP\right)\left(TP+FN\right)\left(TN+FP\right)\left(TN+FN\right)}} \end{array}$$

### 4.3 Quantitative studies

To avoid ambiguity, the module-level experiments in this section are organized under two settings. All comparisons used the same lightweight encoder–decoder backbone, input resolution, optimizer, batch size, number of training epochs, evaluation metrics, and fixed training/validation/test split. The stage-wise data partition described in Section 2 was kept unchanged, and all methods followed the same Early→Middle→Late training order. The stage-specific test sets were not used for training or model selection, and unlabeled samples were used only in the semi-supervised training branch. Therefore, the reported differences mainly reflect the compared module or strategy itself (see Table 1).

For the isolated effectiveness evaluations of NTMC and DCCR, only the target module was enabled, while the other proposed modules were disabled, so that the contribution of each individual module could be assessed directly. The NTMC comparison evaluates different temporal/teacher-learning strategies, and the DCCR comparison evaluates different teacher-update and reliability-control strategies. The vegetation-index input ablation was also conducted under the VCCA-only setting. In contrast, the VCCA fusion-strategy comparison was conducted within the full NDVNet framework, where NTMC and DCCR were kept enabled and only the VCCA fusion block was replaced. This setting was used to evaluate the practical effect of different fusion strategies in the final complete model. Therefore, the VCCA result in the input ablation table corresponds to the VCCA-only setting, whereas the VCCA result in the fusion-strategy table corresponds to the full NTMC + DCCR + VCCA setting. To improve the readability of the main text, detailed module-level comparisons are provided in Supplementary Table S1–S5, while the key component-level ablation and comparison results are retained in Table 2, Table 3, Table 4.

**Table 1:** Implementation details and hyperparameter settings.

Item	Setting	Item	Setting	Item	Setting
Input size	640 × 640	Optimizer	AdamW	Batch size	8
Initial LR	1 × 10^−4^	Weight decay	1 × 10^−4^	Epochs	300
LR scheduler	Cosine decay	Random seed	42	EMA decay	0.90/0.99/0.999
Pseudo-label threshold	0.90/0.98	Maximum consistency weight	1.0	Validation/Test split	10%/15%
Supervised loss	FocalDice	Consistency loss	KL divergence	Reduced consistency weight	0.3

**Table 2:** Component ablation experiment of NDVNet.

NTMC	DCCR	VCCA	mIoU	Precision	F1-score	Recall	MCC
			0.6015	0.6280	0.5117	0.4320	0.5246
✔			0.6236	0.6205	0.5015	0.4208	0.5037
	✔		0.6349	0.6282	0.5119	0.4320	0.5129
		✔	0.6417	0.6335	0.5228	0.4450	0.5186
✔	✔		0.6598	0.6401	0.5319	0.4551	0.5288
	✔	✔	0.6672	0.6485	0.5451	0.4702	0.5369
✔		✔	0.6791	0.6529	0.5374	0.4566	0.5416
✔	✔	✔	0.6829	0.6612	0.5617	0.4882	0.5638

**Table 3:** Comparison experiment results.

Model	mIoU	Precision	MCC	Params (M)	FLOPs (G)
CSL	0.6676	0.5964	0.5107	31.24	185.71
CCVC	0.6374	0.6786	0.4842	29.67	178.36
LVF	0.6313	0.574	0.4751	27.96	165.41
LVR	0.6344	0.583	0.4713	28.64	169.91
Swin-net	0.6544	0.6512	0.4785	41.34	214.85
SegFormer	0.6124	0.6533	0.5068	13.77	65.36
Ours	0.6829	0.6612	0.5638	24.61	142.03

**Table 4:** Results of comparative and generalization experiments.

Group	Dataset	Model	mIoU	F1-score	MCC	Params (M)	FLOPs (G)
	PDT	CSL	0.6815	0.8089	0.5126	31.24	185.71
		CCVC	0.6680	0.7984	0.6735	29.67	178.36
		LVF	0.6681	0.8026	0.7421	27.96	165.41
a		LVR	0.6714	0.8159	0.7486	28.64	169.91
		Swin-net	0.6896	0.8134	0.7027	41.34	214.85
		SegFormer	0.6437	0.7851	0.6849	13.77	65.36
		Ours	0.7142	0.8324	0.7577	24.61	142.03
	Roboflow	CSL	0.6948	0.8169	0.5284	31.24	185.71
		CCVC	0.6726	0.8047	0.6812	29.67	178.36
		LVF	0.6789	0.8086	0.7428	27.96	165.41
b		LVR	0.6817	0.8112	0.7428	28.64	169.91
		Swin-net	0.7012	0.8208	0.7046	41.34	214.85
		SegFormer	0.6524	0.7896	0.6891	13.77	65.36
		Ours	0.7289	0.8443	0.7617	24.61	142.03
	Satellite	CSL	0.6418	0.7685	0.5741	31.24	185.71
		CCVC	0.6129	0.7341	0.6513	29.67	178.36
		LVF	0.6087	0.7489	0.7382	27.96	165.41
c		LVR	0.6115	0.7456	0.7421	28.64	169.91
		Swin-net	0.6294	0.7123	0.6527	41.34	214.85
		SegFormer	0.5863	0.7348	0.6841	13.77	65.36
		Ours	0.6567	0.7502	0.7224	24.61	142.03

#### 4.3.1 Ablation study on the effectiveness of NTMC module

Supplementary Table S1 compares NTMC with representative semi-supervised and teacher–student learning baselines under the same experimental protocol. All methods use the same lightweight encoder–decoder backbone, input resolution, training/validation/test split, training duration, optimizer, and evaluation metrics. Therefore, the performance differences mainly reflect the effect of the compared learning strategies rather than changes in backbone capacity or training budget.

As shown in Supplementary Table S1, NTMC achieves the best overall performance among the compared methods, with mIoU, Precision, F1-score, and MCC of 0.6236, 0.6205, 0.5015, and 0.5037, respectively. The improvement is consistent across multiple metrics rather than being limited to a single score. For PWD canopy segmentation, this is important because a higher mIoU alone may result from over-expanded masks. The simultaneous improvement in F1-score and MCC indicates that NTMC improves lesion delineation while maintaining more reliable foreground–background discrimination.

Supplementary Table S1compares NTMC with representative teacher–student and semi-supervised segmentation baselines, including Mean Teacher [32]; FixMatch [33]; FlexMatch [34]; CPS [21]; U2PL [35].

To further clarify whether the improvement of NTMC simply comes from using multiple EMA teachers, we conduct an internal ablation study by progressively decomposing NTMC into several variants. Specifically, Single EMA teacher corresponds to a conventional one-teacher EMA setting. Fixed multi-EMA introduces fast, medium, and slow EMA teachers with fixed update rates. Drift-aware multi-EMA further allows the update rhythm to change according to the training drift. Nested adaptive EMA adds the monotonic fast–medium–slow temporal constraint. Full NTMC additionally introduces agreement-based reliability control among the multi-tempo predictions. All variants follow the same Early→Middle→Late training order and use the same backbone, data split, optimizer, training epochs, and evaluation metrics.

As shown in Supplementary Table S2, Fixed multi-EMA improves over Single EMA teacher, indicating that maintaining multiple temporal trajectories can provide more stable teacher targets than a single smoothed trajectory. However, the improvement remains limited, suggesting that simply increasing the number of EMA teachers is insufficient to fully address stage-wise drift. After introducing drift-aware updates, the performance further improves, showing that adaptive update rhythms are beneficial for sequential PWD phenotype learning. Nested adaptive EMA obtains additional gains by enforcing an ordered fast–medium–slow temporal hierarchy, which helps balance current-stage adaptation and previous-stage retention. Finally, Full NTMC achieves the best results across all four metrics. This indicates that the performance gain of NTMC is not merely caused by multi-teacher ensemble, but mainly comes from the combination of multi-tempo trajectories, drift-aware updates, nested temporal constraints, and agreement-based reliability control.

#### 4.3.2 Ablation study on the effectiveness of DCCR module

Supplementary Table S3 compares DCCR with representative teacher-update and consistency-learning variants under the same experimental protocol. All variants use the same student backbone, input resolution, training/validation/test split, training duration, optimizer, and evaluation metrics. Therefore, the performance differences mainly reflect the influence of the teacher-update strategy and reliability-control mechanism, rather than differences in backbone capacity or training budget.

As shown in Supplementary Table S3, DCCR achieves the best overall performance, with mIoU, F1-score, and MCC of 0.6349, 0.5119, and 0.5129, respectively. Although SWA-Teacher obtains the highest Precision, its mIoU, F1-score, and MCC remain much lower, indicating that high precision alone does not lead to balanced diseased-canopy segmentation. Compared with the other teacher variants, DCCR provides more consistent improvements across multiple metrics, suggesting that reliability-aware consistency learning is more effective for suppressing noisy pseudo-label supervision in PWD canopy segmentation, especially under blurred lesion boundaries and complex forest backgrounds.

Supplementary Table S3compares DCCR with representative teacher-update and semi-supervised consistency-learning variants, including SWA-Teacher[36]; Cosine-EMA Teacher; Dual-EMA Teacher(Fast/Slow)[37]; MC-Dropout EMA Teacher [38], and Lookahead Teacher[39].

**Fig. 3:**
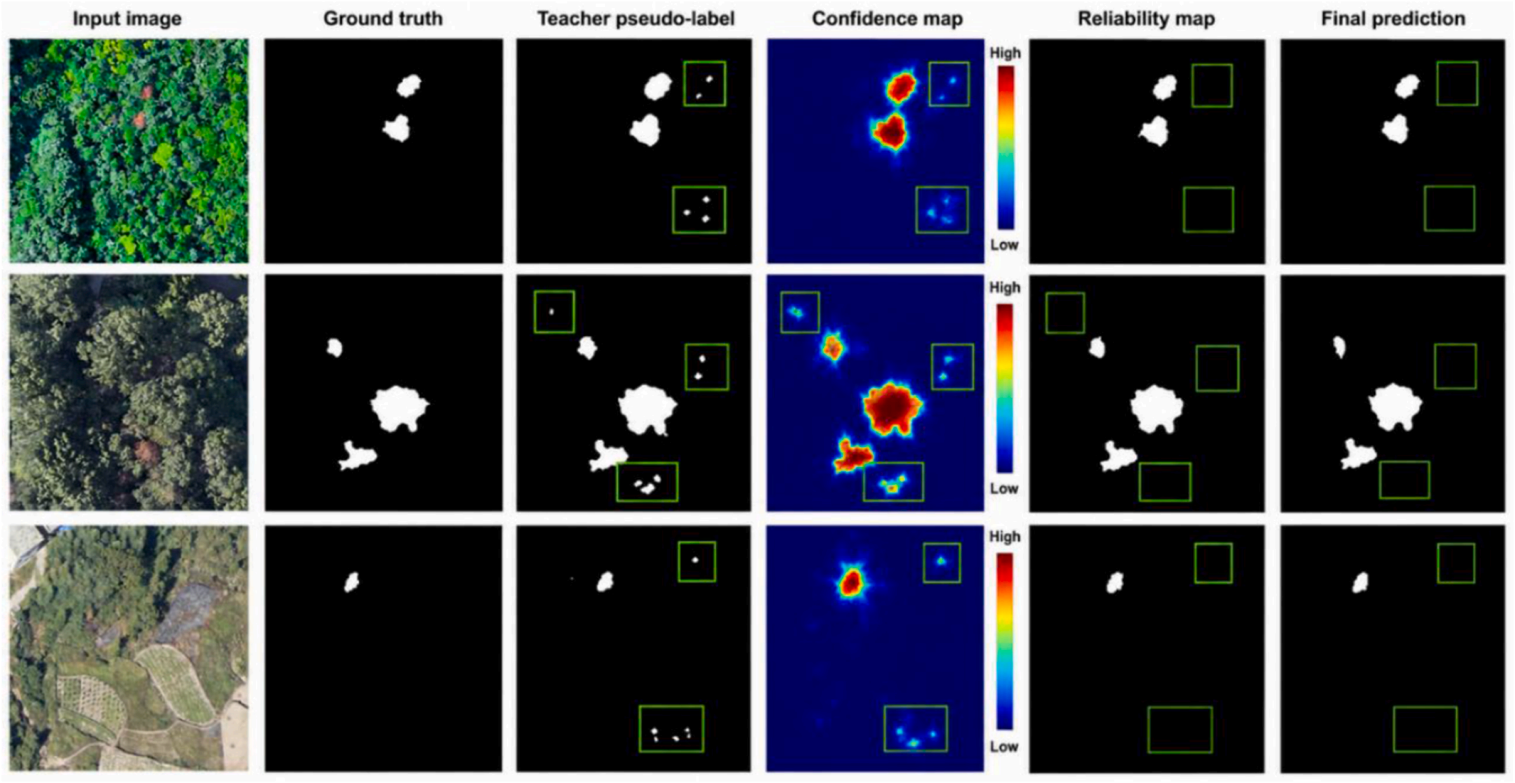
Visualization of pseudo-label reliability control in DCCR. From left to right are the input image, ground truth, teacher pseudo-label, confidence map, reliability map, and final prediction. Green boxes highlight ambiguous or noisy regions in the teacher pseudo-labels. The reliability map down-weights or suppresses these low-confidence regions during consistency learning, leading to cleaner final predictions with fewer false activations.

To further explain why DCCR improves semi-supervised learning, we visualize the pseudo-label reliability control process in Fig. 3. The teacher pseudo-labels contain uncertain or noisy responses around blurred lesion boundaries, weak-symptom regions, and texture-similar backgrounds. The confidence maps reveal these ambiguous regions, while the reliability maps suppress low-confidence responses and preserve more reliable diseased-canopy cues for consistency learning. As a result, the final predictions show fewer false activations and cleaner lesion shapes than the raw teacher pseudo-labels. This visualization supports the quantitative results in Supplementary Table S3 and indicates that DCCR improves segmentation mainly by reducing the propagation of unreliable pseudo-label supervision.

#### 4.3.3 Ablation study on the effectiveness of VCCA module

To quantify the contribution of vegetation-index information, we first conduct an RGB-only control experiment under the same backbone and training protocol, where VI cues are removed and VCCA conditioning is disabled. As shown in Supplementary Table S4, the RGB-only baseline achieves an mIoU of 0.6015. After introducing a single vegetation index, mIoU increases to 0.6306 with EVI and 0.6234 with NDVI, indicating that vegetation-index cues provide useful physiological information beyond RGB appearance.

In addition to NDVI and EVI, we also include GNDVI and SAVI as commonly used vegetation-index references for comparison. Their definitions are given as follows:

(22) $$GNDVI=\frac{\rho_{NIR}-\rho_{G}}{\rho_{NIR}+\rho_{G}+\epsilon },SAVI=\frac{\left(1+L\right)\left(\rho_{NIR}-\rho_{R}\right)}{\rho_{NIR}+\rho_{R}+L+\epsilon }\text{.}$$

Compared with the RGB-only baseline, GNDVI and SAVI also improve mIoU, but their gains are smaller, reaching 0.6158 (+1.43 percentage points) and 0.6085 (+0.70 percentage points), respectively. Combining EVI and NDVI further increases mIoU to 0.6417 (+4.02 percentage points), suggesting that these two indices provide complementary physiological cues and support the effectiveness of VI-guided calibration in VCCA.

To systematically examine how vegetation-index information should be exploited for segmentation, we compare five representative fusion baselines with the proposed VCCA design under the same backbone and training protocol. In these experiments, all variants are evaluated within the complete framework, where NTMC and DCCR are kept enabled, and only the VCCA-related fusion block is replaced by different vegetation-index fusion designs. This setting ensures that the observed performance differences mainly reflect the effect of the fusion strategy itself rather than changes in the backbone, training schedule, or data partitioning.

The compared strategies include multi-source parallel fusion, gated selection, cross-modal interaction, redundancy-reduced fusion, and the proposed VCCA design. These settings are used to examine whether reliable vegetation-index responses can be effectively selected and whether redundancy or conflict among multiple index cues can be reduced during RGB–VI interaction. As shown in Supplementary Table S5, different fusion strategies show different performance preferences. Parallel soft-orthogonal fusion achieves the highest mIoU, suggesting slightly better region overlap. However, VCCA obtains the best Precision, F1-score, and MCC while maintaining a competitive mIoU, indicating a more balanced trade-off between lesion-region overlap, foreground reliability, and overall segmentation consistency.

### 4.4 Ablation studies

To avoid drawing conclusions from single-module comparisons only, we further evaluate pairwise combinations and the full setting, as shown in Table 2. Among the two-module settings, NTMC + DCCR improves mIoU and F1-score to 0.6598 and 0.5319, respectively. This suggests that stage-aware temporal consolidation can make reliability-controlled pseudo supervision more stable across infection stages. DCCR + VCCA achieves a balanced improvement (mIoU = 0.6672, F1-score = 0.5451, MCC = 0.5369), indicating that reliability filtering works more effectively when the feature representation is supported by vegetation-index cues. NTMC + VCCA obtains performance close to the full model (mIoU = 0.6791, Precision = 0.6529, F1-score = 0.5374, MCC = 0.5416), which suggests that stage-wise knowledge retention becomes easier when RGB features are calibrated by physiologically relevant NDVI/EVI information.

These results also explain why the three modules are complementary rather than redundant. VCCA first improves feature discrimination by introducing vegetation-index-guided physiological cues, which helps reduce confusion between diseased canopy regions and visually similar backgrounds. DCCR then suppresses unreliable pseudo-label supervision in ambiguous boundary or weak-symptom regions, reducing the risk of error accumulation during semi-supervised learning. NTMC further stabilizes the learning process by maintaining fast, medium, and slow temporal trajectories, which helps preserve early- and middle-stage phenotypic information when the model is exposed to later-stage samples. With all three modules enabled, the model achieves the best overall profile (mIoU = 0.6829, Precision = 0.6612, F1-score = 0.5617, MCC = 0.5638), showing that the final improvement comes from the interaction among feature calibration, pseudo-label reliability control, and cross-stage memory consolidation.

It is worth noting that NTMC alone improves mIoU from 0.6015 to 0.6236, while Precision, Recall, F1-score, and MCC decrease slightly. This behavior suggests that NTMC mainly enhances region-level overlap by preserving stage-dependent representations during sequential learning. However, when used in isolation, the resulting predictions may become less balanced between lesion coverage and foreground–background discrimination. Similarly, DCCR-only and VCCA-only improve mIoU but do not consistently improve MCC. This indicates that each module mainly addresses a specific aspect of the problem rather than optimizing all evaluation criteria simultaneously. DCCR focuses on reducing unreliable pseudo-supervision, while VCCA strengthens physiology-related feature responses through vegetation-index guidance. When combined, the complementary effects of NTMC, DCCR, and VCCA lead to consistent improvements across all metrics, demonstrating that the full performance gain originates from their interaction rather than any individual module alone.

### 4.5 Comparative studies

Table 3 reports the quantitative results on our self-constructed dataset. The compared methods are broadly able to segment diseased crowns, but they differ mainly in how they balance lesion coverage against decision reliability under cluttered backgrounds and rare positives. CSL reaches a higher mIoU of 0.6676 but a lower MCC of 0.5107, whereas CCVC shows another trade-off (mIoU 0.6374, MCC 0.4842). Our method ranks best overall, achieving 0.6829 mIoU and 0.5638 MCC with moderate complexity (24.61M parameters and 142.03G FLOPs). Detailed interpretation is left to the Discussion.

Table 3 reports the comparison experiment results on our self-constructed dataset. The evaluated methods include CSL [26]; CCVC [40]; LVF [41]; LVR [42]; Swin-net [43]; SegFormer [44], “Ours” denotes our proposed method.

**Fig. 4:**
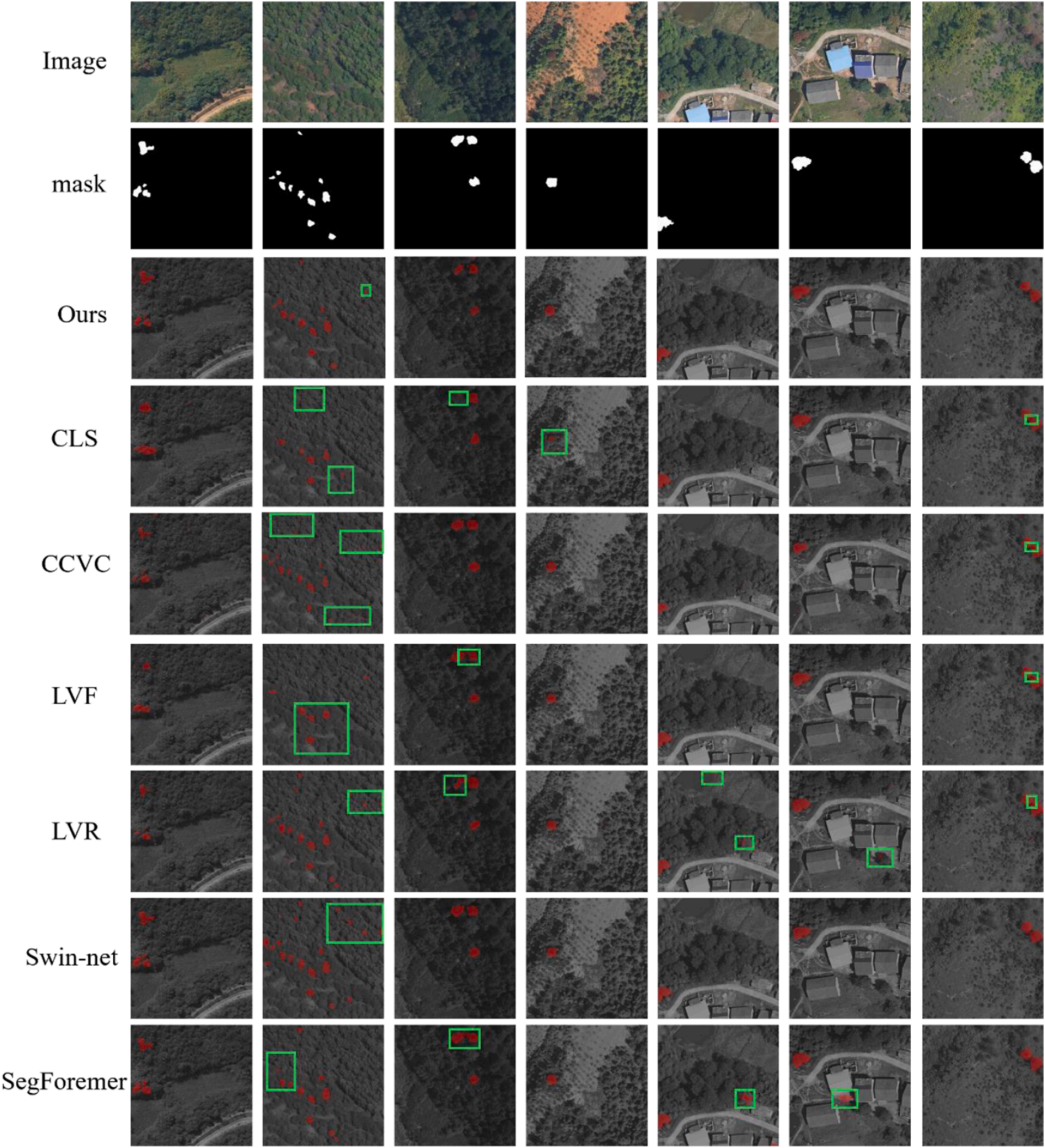
Qualitative visualization corresponding to the comparison results in Table 3. The red regions denote the predicted diseased-canopy masks. The green boxes are manually added visual markers used to highlight representative incorrectly segmented regions, including missed segmentation, false segmentation, and boundary mismatch. It should be noted that the green boxes are not model-generated detection boxes, but are only used to illustrate the segmentation differences among different methods in challenging regions.

Qualitative examples are shown in Fig. 4. The red regions indicate the predicted masks, while the green boxes highlight areas where the predictions do not match the ground truth labels. In these challenging scenes with dense canopy texture, nearby built-up structures, and weak-contrast crowns, the mismatches mainly appear as boundary misalignment around diffuse lesion edges, as well as local missing or extra predicted fragments in texture-confusing areas. Compared with the baselines, our results show fewer obvious mismatches within the highlighted regions and produce more coherent lesion shapes, especially when background interference is strong and lesion cues are weak.

### 4.6 Generalization performance

The PDT dataset is a high-precision UAV pest-and-disease tree dataset (captured at an altitude of ∼200 m, with complex scenes and fine-grained annotations). We select 400 PWD images from it for generalization experiments. The Roboflow PWD dataset is a public benchmark containing 1000 samples from multiple regions and infection stages (split into 700/150/150). In addition, we use an externally obtained satellite remote-sensing dataset for out-of-domain generalization testing to further examine the model's generalization capability under cross-scenario and cross-platform distribution gaps. All these generalization experiments follow a semi-supervised setting with a small amount of labeled data and a large amount of unlabeled data.

In Table 4, groups a, b, and c report the results on the PDT, Roboflow, and satellite datasets, respectively.

**Fig. 5:**
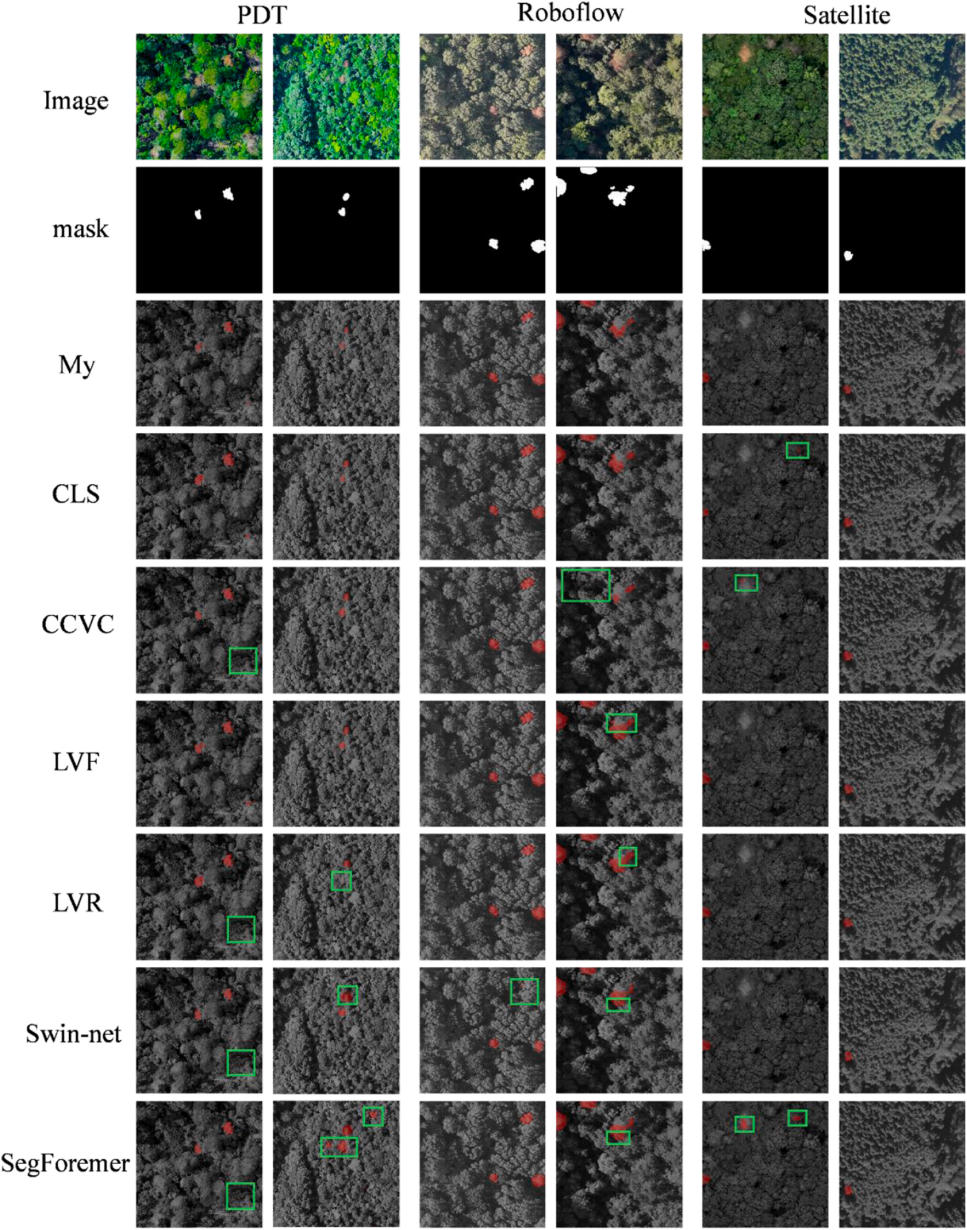
Qualitative visualization corresponding to the generalization results in Table 4. The red regions denote the predicted diseased-canopy masks on different external datasets. The green boxes are manually added visual markers used to highlight representative missed or incorrectly segmented regions. This figure is provided for intuitive comparison of cross-dataset generalization performance, while the quantitative accuracy is reported in Table 4 using mIoU, F1-score, and MCC.

On the PDT dataset, all baseline methods demonstrate useable segmentation capability, and the differences mainly lie in whether informative cues are retained while noise is suppressed at the same time. Compared with the non-lightweight Swin-net (mIoU = 0.6896, 41.34M parameters, 214.85G FLOPs) and conventional semi-supervised/multimodal baselines (e.g., CSL, CCVC, LVF, and LVR), Ours achieves the best results in terms of mIoU/F1-score/MCC (0.7142/0.8324/0.7577), with a more moderate complexity (24.61M parameters, 142.03G FLOPs). This indicates that, under a public-domain distribution, the proposed method can more effectively extract stable index cues correlated with lesions and reduce the accumulation of spurious responses caused by background textures of similar appearance, thereby improving overlap quality and global consistency simultaneously, rather than trading one metric off to obtain a single-point gain. The quantitative results are summarized in Table 4, and representative visual comparisons are provided in Fig. 5.

On the Roboflow dataset, the contrast in generalization robustness becomes more visible. Several methods keep F1-score at a reasonable level, but their mIoU and MCC improve only slightly, which is often a sign that masks remain unstable around crown edges and small local errors accumulate when the domain changes. In comparison, Ours reaches mIoU = 0.7289, F1-score = 0.8443, and MCC = 0.7617, the best results on this benchmark, while still requiring much less computation than heavy backbones such as Swin-net. In practical canopy mapping terms, this means the model is not only finding diseased regions, but also keeping the crown structure more coherent, with fewer false activations from background patterns that resemble stress signals.

On the satellite dataset, performance drops for most methods, indicating a stronger domain shift between satellite imagery and UAV imagery. To further analyze the main sources of this degradation, we conducted a test-time degradation sensitivity analysis by simulating resolution loss, scale difference, illumination variation, and atmospheric-like disturbance. As shown in Supplementary Table S6, the original UAV test performance reaches 0.6829 mIoU, 0.5617 F1-score, and 0.5638 MCC. Among the simulated degradation factors, the 0.5×target-scale setting causes the largest decrease, with drops of 0.0761, 0.0748, and 0.0712 in mIoU, F1-score, and MCC, respectively. The 0.5×downsampling-and-restoration setting leads to the second-largest decrease, with drops of 0.0613, 0.0593, and 0.0551. In contrast, brightness/contrast perturbation and haze-like blur result in relatively smaller decreases.

These results suggest that the performance drop on the satellite dataset is more likely dominated by scale differences and resolution loss. Compared with UAV imagery, satellite imagery provides coarser spatial details, and diseased-canopy regions occupy fewer pixels. Therefore, small lesion regions and gradual canopy boundaries are more easily compressed, blurred, or mixed with the background, leading to under-segmentation, boundary shrinkage, and reduced region-level overlap. Atmospheric and illumination effects can still affect RGB appearance and vegetation-index responses, but they appear to further aggravate feature instability rather than dominate the degradation. Even under this stronger domain shift, our method still achieves the highest mIoU on the satellite dataset, indicating its advantage in region-level overlap and efficiency. Future improvements should focus more on satellite-specific adaptation strategies, such as scale-aware feature modeling, resolution enhancement, vegetation-index normalization, and boundary-consistency constraints.

## 5 Discussion

### 5.1 Experimental results discussion

**Fig. 6:**
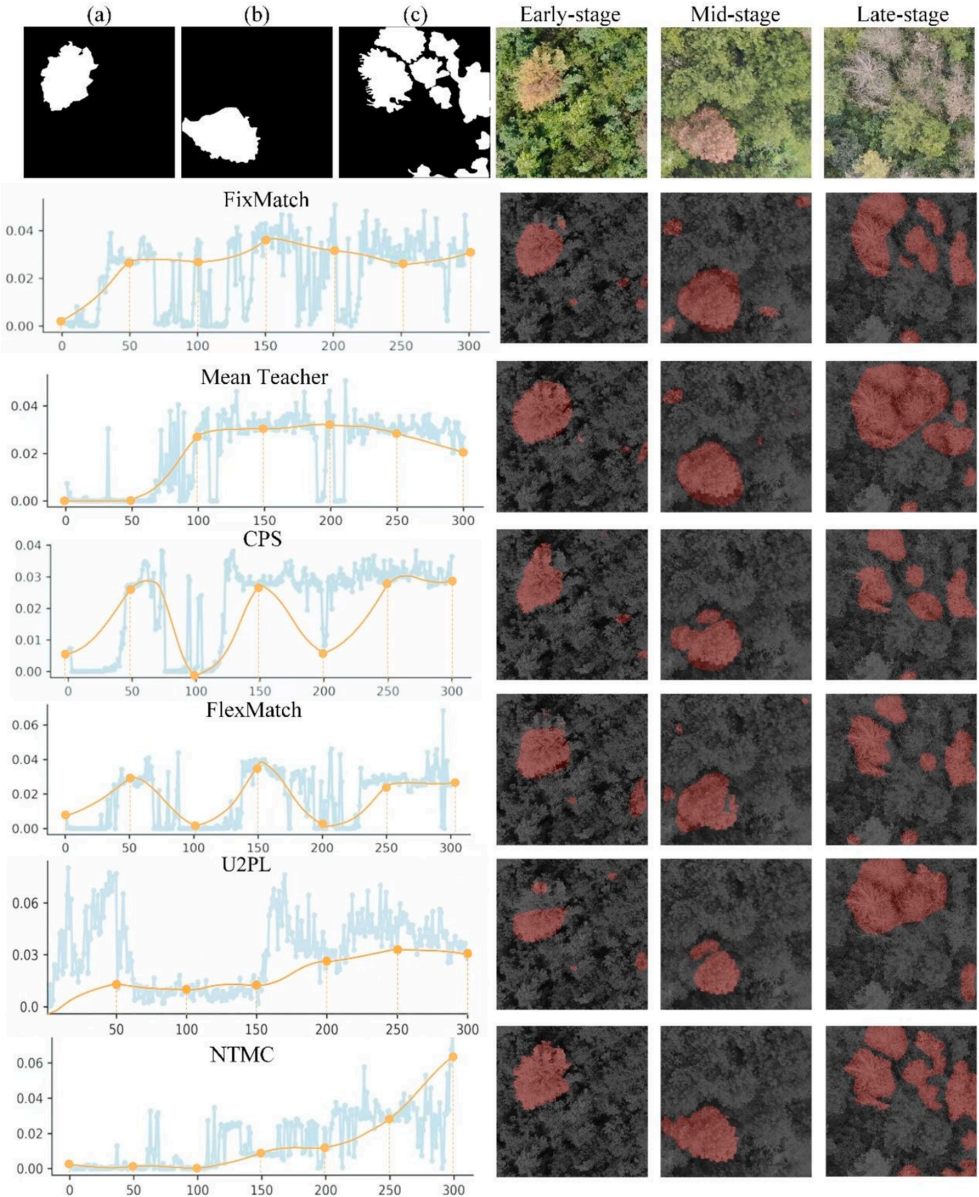
Cross-stage phenotypic consolidation curves and qualitative visualizations. Panels (a), (b), and (c) correspond to the labels of early-stage, middle-stage, and late-stage infections, respectively.

The effectiveness results in Supplementary Tables S1, S3, and S5 suggest that the three proposed components improve performance through different mechanisms, rather than the same shortcut. For NTMC, the key signal is the consistent gain across mIoU, Precision, F1-score, and MCC, instead of an isolated advantage in a single score. In this task, positives are sparse and lesion boundaries are often gradual, so overlap can increase simply because predictions expand. When the improvement comes together with F1-score and MCC, it more plausibly indicates more reliable foreground–background decisions under texture interference, rather than mask expansion alone. This is why NTMC is revisited in the subsequent cross-stage phenotypic consolidation analysis. Fig. 6 links the consolidation curves with cross-stage visualizations, providing a training-dynamics view of whether the observed gains correspond to steadier stage transitions and more consistent early/middle/late predictions. These results also clarify the methodological position of NTMC. NTMC should not be interpreted as a completely independent learning paradigm separated from EMA teacher–student learning. Instead, it is a task-specific extension that restructures EMA trajectories into a drift-aware nested tempo hierarchy for stage-wise PWD segmentation.

For DCCR, Supplementary Table S3 shows that “changing the teacher” alone often pushes the method toward extreme behavior. Some variants become overly cautious, producing cleaner positives but missing large fractions of diseased crowns; others gain overlap mainly by loosening specificity, which is risky when background textures mimic stress patterns. The underlying issue is that PWD canopy symptoms are weak and transitional, so pseudo-labels are easy to contaminate near boundaries and in cluttered scenes. DCCR is valuable because it explicitly couples reliability calibration with consistency, turning unlabeled data into training signal only when it is less likely to amplify these errors. This explains why it behaves differently from teacher-update heuristics: the bottleneck here is not “how smooth the teacher is,” but whether the pseudo supervision is trustworthy in hard regions.

The VCCA results in Supplementary Tables S4 and S5 indicate that vegetation-index cues are helpful, but the way they are combined matters more than simply “adding more indices.” In canopy phenotyping, index responses can be locally unstable due to illumination, mixed pixels, and background materials; naive aggregation may therefore propagate noise, while overly aggressive interaction can introduce redundant resonance and conflict diffusion. The grouped strategies proposed in this work are informative because they separate two mechanisms: selecting reliable index responses before fusion, and controlling redundancy/conflicts when multiple sources interact. VCCA is then best understood as a design choice that prioritizes stable, physiology-linked cues that remain compatible with semi-supervised consistency learning, which becomes clearer when it is placed into the full model and evaluated with other components.

Ablations in Table 2 further clarify how these roles complement each other. Single-module gains indicate that each component addresses a distinct failure mode: VCCA tends to strengthen lesion-related cues, DCCR controls error propagation from pseudo-supervision, and NTMC is more about keeping cross-stage learning from drifting. The pairwise settings make this easier to interpret. DCCR + VCCA yields a steadier improvement profile because reliability control and index-guided cues naturally reinforce each other in cluttered scenes, while NTMC + VCCA coming close to the best configuration is consistent with a simple intuition: when the representation is already better anchored by physiology-linked cues, stage-wise consolidation becomes easier to exploit. The full model performs best not because one module dominates, but because the pipeline closes the loop: cue quality improves, unreliable pseudo-label signals are suppressed, and cross-stage consolidation is less frequently disrupted. This also sets up the next section, where NTMC's contribution is examined from a training-dynamics perspective rather than only through endpoint metrics.

The comparison in Table 3 and the qualitative figure (red masks as predictions, green boxes marking mismatches with annotations) reflects a common pattern for rare-positive segmentation under heavy background interference. Methods may look similar in “whether they can segment lesions at all,” yet diverge in how they trade lesion coverage against decision reliability. It is easy to obtain higher overlap by allowing predictions to spread in ambiguous regions, but this typically increases annotation mismatches in the boxed areas and weakens MCC. Conversely, conservative behavior reduces false activations but tends to miss faint or fragmented lesions. Our results are strongest when judged by the combined profile (mIoU and MCC, and moderate complexity), which aligns with the visual goal in canopy mapping: not only finding diseased crowns, but keeping masks coherent and less sensitive to texture-confusing backgrounds. The detailed error types are intentionally not over-interpreted here, since the next discussion focuses on what causes instability and how it accumulates across stages.

Generalization results in Table 4 are consistent with the above interpretation. On UAV-domain benchmarks (PDT and Roboflow), the proposed method maintains the best overall scores while staying lighter than heavy backbones, suggesting that its advantage is not tied to a specific dataset idiosyncrasy. The reason this carries across UAV datasets is plausible: physiology-linked cues remain informative, and reliability-controlled pseudo-supervision reduces the chance that background textures are repeatedly reinforced as positives. The satellite domain introduces a stronger shift—resolution and scale change, illumination and surface materials differ, and index responses can be less stable—so a universal drop is expected. The split ranking there is also meaningful: some methods can look good on F1-score or MCC alone by adopting a particular trade-off, while region-level overlap (mIoU) becomes harder to preserve. That Ours retains the best mIoU under this hardest shift suggests better region-level alignment, while the remaining gap indicates that satellite-specific handling (e.g., index normalization and boundary consistency under coarse resolution) is the more relevant direction than simply enlarging the backbone.

### 5.2 Cross-stage phenotypic consolidation analysis

To simulate the dynamic progression of pine wilt disease (PWD) phenotypes in real monitoring scenarios, we formulate training as a staged sequential task. Unlike conventional static segmentation that treats all samples as a single fixed task, our setting requires the model to adapt continuously under stage-wise distribution shifts while retaining previously learned early-stage cues. Concretely, we partition the dataset into three subsets according to disease severity (early, middle, and late), and train the model sequentially in the biological order (Early→Middle→Late) with stage-wise batches (Stages/Epochs). This protocol is designed to evaluate whether the model can consolidate subtle early-stage phenotypic memory when exposed to more salient late-stage symptoms, thereby mitigating catastrophic forgetting. Based on this staged cross-stage training protocol, we further visualize and analyze training-time stabilization via cross-stage phenotypic consolidation curves, as shown in Fig. 6.

As shown in Fig. 6, the top row presents representative early-stage, middle-stage, and late-stage PWD canopy samples together with their corresponding annotations (a–c). The blue curves below report the cross-stage phenotypic consolidation curves of different methods, where the x-axis denotes training epochs and the y-axis reflects the accumulated knowledge consolidation strength during training. Formally, we define the consolidation strength as a cumulative, reliability-weighted agreement between the student prediction and the cross-stage ensemble target on unlabeled pixels. Specifically, at epoch e, the instantaneous consolidation score is computed as:

(23) $$s_{e}=\frac{\sum_{i\in \Omega }M_{i}r_{i}\left(1-\tilde{D}\left(p_{\theta }{\left(A_{s}\left(x_{u}\right)\right)}_{i},Q_{i}\right)\right)}{\sum_{i\in \Omega }M_{i}+\varepsilon }\text{.}$$

and the accumulated consolidation strength up to epoch E is

(24) $$\text{CS}\left(E\right)=\sum_{e=1}^{E}s_{e}\text{.}$$

Here, $p_{\theta }\left(A_{s}\left(x_{u}\right)\right)i$ denotes the student prediction at pixel i under strong augmentation, $Q_{i}$ is the stop-gradient multi-tempo ensemble target, $M_{i}$ is the reliability-gating mask, and $r_{i}$ is the agreement-based reliability weight. $\tilde{D}\left(\cdot ,\cdot \right)$ is a normalized discrepancy (instantiated by the same criterion l(·) used in LNTMC, e.g., KL divergence or MSE, scaled to [0,1]), and Ω denotes the pixel set. This quantity can be interpreted as the cumulative stabilization invested in the learned phenotypic representations; in general, a smoother and more steadily increasing curve suggests a more coherent consolidation process with less disruption from stage transitions. On the right, the corresponding segmentation visualizations are provided to examine whether the curve trends are reflected in stable cross-stage segmentation performance. Most competing methods show clear stage sensitivity. Around several milestones, frequent and large spikes appear, indicating that their consolidation is easily disturbed by stage switching and tends to oscillate afterward. FlexMatch and CPS exhibit a typical periodic “rise–drop–rise” pattern, suggesting intermittent instability during consolidation. U2PL remains relatively smoother in the mid stage, but its late-stage curve becomes more volatile and its growth rate less stable, implying higher sensitivity to distribution shifts in the later phase. By contrast, NTMC yields a more coherent trajectory: spikes at stage transitions are fewer, recovery is faster, and the late-stage curve keeps rising before gradually stabilizing, indicating that NTMC achieves cross-stage memory alignment and progressive consolidation without relying on aggressive “compensatory” updates.

The segmentation visualizations on the right help verify whether training stability translates into cross-stage prediction stability. When the curve becomes unstable near certain milestones, the corresponding stage samples often show fragmented boundaries, local under-segmentation, or increased false activations in the background. When the curve remains steadily increasing across stages, predictions on early/middle/late samples are more consistent, with more complete lesion coverage, more coherent shapes, and fewer spurious responses to complex backgrounds. This consistency between “smoother curves” and “more stable cross-stage masks” indicates that the method not only consolidates knowledge more reliably during training, but also delivers stronger stage-wise generalization in the resulting segmentations.

Although NTMC demonstrates a more stable consolidation trend in cross-stage training, some cases still exhibit insufficient fine-grained delineation. This limitation likely stems from the object itself: phenotypic differences across stages can be substantial, lesion boundaries are often gradual rather than sharp, and complex backgrounds can make a small portion of hard cases inherently ambiguous. In future work, we will refine the feature extraction design with stage-aware emphasis on boundary characteristics to further improve segmentation precision for PWD canopies.

## 6 Conclusions

We constructed a standardized UAV-based canopy segmentation dataset for pine wilt disease with support from the Hunan Academy of Forestry. The dataset contains pixel-level annotations and unlabeled samples across early, middle, and late infection stages, providing a practical basis for stage-aware and semi-supervised PWD canopy segmentation. Based on this dataset, we developed NDVNet to address three practical difficulties in PWD canopy phenotyping: stage-wise representation drift, unreliable pseudo-label supervision, and RGB-based background confusion.

First, stage-wise phenotypic changes can cause the model representation to drift toward later-stage symptoms and weaken subtle early-stage cues. NTMC alleviates this issue by maintaining nested fast/medium/slow temporal trajectories, which helps retain useful information from different infection stages during sequential learning. Second, fine-grained canopy masks are expensive and may be affected by blurred boundaries and annotation variability. DCCR improves the use of unlabeled images by applying reliability-weighted consistency learning, reducing the influence of unreliable pseudo-labels in ambiguous regions. Third, RGB appearance alone is often insufficient for distinguishing diseased canopies from texture-similar forest backgrounds. VCCA incorporates NDVI and EVI as physiological cues to calibrate RGB features, improving the discrimination of diseased canopy regions.

The three components are complementary in our experiments. On the in-house dataset, the full model achieves the best overall results, with mIoU = 0.6829, Precision = 0.6612, F1-score = 0.5617, and MCC = 0.5638, improving over the baseline model while maintaining moderate computational complexity. The ablation and comparative experiments show that NDVNet improves lesion-region delineation, pseudo-label reliability, and foreground–background discrimination. Experiments on external datasets further indicate its potential for practical UAV-based forest disease monitoring.

In future work, we will explore additional physiology-sensitive modalities and model compression strategies to improve deployment efficiency for UAV-based monitoring scenarios.

## Author contributions

Tao Liu and Wentao Peng contributed equally to this work. Tao Liu and Wentao Peng: Conceptualization, Methodology, Software, Formal analysis, Investigation, Writing—original draft. Huaiqing Zhang, Lin Hui, Langwen Tang, Jingdong Li, Xinyu Zhang, Chaoying He, and Zilin Ye: Data curation, Validation, Visualization. Yingfang Zhu, Guoxiong Zhou, and Shichao Jin: Supervision, Resources, Project administration, Writing—review and editing. All authors have read and agreed to the published version of the manuscript.

## Funding

This research was supported by the Fundamental Research Fund for Central Public Interest Scientific Institution under Grant No. 1630032022007, the National Key R&D Program of China under Grant No. 2019YFD1000500, and the Central Forestry and Grassland Reform and Development Fund (Hunan Major Forestry and Grassland Pest Prevention and Information-based Monitoring).

## Declaration of competing interest

The author is an Editorial Board Member/Editor-in-Chief/Associate Editor/Guest Editor for this journal and was not involved in the editorial review or the decision to publish this article.

The authors declare the following financial interests/personal relationships which may be considered as potential competing interests:Given his role as Senior Editor, Shichao Jin had no involvement in the peer review of this article and had no access to information regarding its peer review. Full responsibility for the editorial process for this article was delegated to another journal editor.

## Data Availability

The in-house UAV canopy dataset used in this study was provided by the Hunan Academy of Forestry. Due to data management and sharing restrictions, the full dataset is not publicly available, but may be made available from the corresponding authors upon reasonable request and with permission from the data provider. The trained model weights, lightweight testing code, and a subset of sample data for evaluation and review are publicly available at: https://github.com/LT-12345/NDVNet-PWD.git. Public benchmark datasets used for comparison are available from their original sources as cited in the manuscript.
