# Supplementary material for: A UAV-based semi-supervised segmentation framework for pine wilt disease via vegetation Index–RGB synergy
Source: Plant Phenomics. 2026 Jun 27;8(3):100255. doi: 10.1016/j.plaphe.2026.100255 (PMC13452197; doi:10.1016/j.plaphe.2026.100255)
Supplement: Supplementary file 1 [file mmc1.docx]

**Appendix S. Detailed module-level and degradation analyses**

To keep the main text concise, the detailed module-level comparisons and the degradation sensitivity analysis are provided below. These appendix tables correspond to the table citations in Sections 4.3–5.1.

**Table S1**
Effectiveness evaluation of NTMC.

| Model | mIoU | Precision | F1-score | MCC |
| --- | --- | --- | --- | --- |
| Mean Teacher | 0.5984 | 0.6042 | 0.474 | 0.4821 |
| FixMatch | 0.6067 | 0.611 | 0.4862 | 0.491 |
| FlexMatch | 0.6179 | 0.6158 | 0.4937 | 0.4979 |
| CPS | 0.6125 | 0.6189 | 0.4989 | 0.5012 |
| U2PL | 0.6208 | 0.6196 | 0.5003 | 0.5026 |
| NTMC | 0.6236 | 0.6205 | 0.5015 | 0.5037 |

**Table S2**
Internal ablation study of NTMC.

| **Variant** | **mIoU** | **Precision** | **F1-score** | **MCC** |
| --- | --- | --- | --- | --- |
| Single EMA teacher | 0.5984 | 0.6042 | 0.474 | 0.4821 |
| Fixed multi-EMA | 0.6062 | 0.6098 | 0.4857 | 0.4908 |
| Drift-aware multi-EMA | 0.6138 | 0.6152 | 0.4931 | 0.4967 |
| Nested adaptive EMA | 0.6195 | 0.6186 | 0.4984 | 0.501 |
| Full NTMC | 0.6236 | 0.6205 | 0.5015 | 0.5037 |

**Table S3**
Effectiveness evaluation of DCCR.

| Model | mIoU | Precision | F1-score | MCC |
| --- | --- | --- | --- | --- |
| SWA-Teacher | 0.5832 | 0.6706 | 0.3009 | 0.3571 |
| Cosine-EMA Teacher | 0.6021 | 0.4584 | 0.3562 | 0.3595 |
| Dual-EMA Teacher(Fast/Slow) | 0.5538 | 0.5838 | 0.2125 | 0.2718 |
| MC-Dropout EMA Teacher | 0.5633 | 0.3362 | 0.2465 | 0.2491 |
| Lookahead Teacher | 0.5808 | 0.5772 | 0.2947 | 0.3337 |
| DCCR | 0.6349 | 0.6282 | 0.5119 | 0.5129 |

**Table S4**
Ablation study of vegetation-index inputs for VCCA.

| Input | mIoU | Precision | F1-score | Recall | MCC |
| --- | --- | --- | --- | --- | --- |
| RGB | 0.6015 | 0.628 | 0.5117 | 0.432 | 0.5246 |
| RGB+GNDVI | 0.6158 | 0.631 | 0.5164 | 0.4371 | 0.5215 |
| RGB+SAVI | 0.6085 | 0.6292 | 0.5137 | 0.4341 | 0.5201 |
| RGB+NDVI | 0.6234 | 0.6326 | 0.5190 | 0.4415 | 0.5225 |
| RGB+EVI | 0.6306 | 0.6331 | 0.5206 | 0.4423 | 0.5232 |
| RGB+EVI+NDVI | 0.6417 | 0.6335 | 0.5228 | 0.4450 | 0.5186 |

**Table S5**
Effectiveness evaluation of VCCA.

| Model | mIoU | Precision | F1-score | MCC |
| --- | --- | --- | --- | --- |
| parallel | 0.6820 | 0.6243 | 0.544 | 0.5439 |
| Series_ndvi_gate | 0.6847 | 0.6224 | 0.5496 | 0.5447 |
| Series_evi_gate | 0.6695 | 0.6015 | 0.5174 | 0.5177 |
| Parallel_cross_mix | 0.6844 | 0.6585 | 0.5484 | 0.5546 |
| Parallel_soft_orthogonal | 0.6869 | 0.6436 | 0.5539 | 0.5548 |
| VCCA | 0.6829 | 0.6612 | 0.5617 | 0.5638 |

**Table S6**
Sensitivity analysis of different degradation factors.

| **Test setting** | **Main simulated factor** | **mIoU** | **F1-score** | **MCC** |
| --- | --- | --- | --- | --- |
| Original UAV test set | None | 0.6829 | 0.5617 | 0.5638 |
| 0.5× downsampling and restoration | Resolution loss | ↓0.0613 | ↓0.0593 | ↓0.0551 |
| 0.5× target scale | Scale difference | ↓0.0761 | ↓0.0748 | ↓0.0712 |
| Brightness/contrast perturbation | Illumination variation | ↓0.0295 | ↓0.0265 | ↓0.0242 |
| Haze-like blur | Atmospheric-like effect | ↓0.0402 | ↓0.0389 | ↓0.0344 |

Values in the first row denote the original UAV test performance, while downward arrows indicate the absolute performance decrease under each simulated degradation setting.
